# Depletion of gut microbiota induces skeletal muscle atrophy by FXR-FGF15/19 signalling

**DOI:** 10.1080/07853890.2021.1900593

**Published:** 2021-03-30

**Authors:** Yixuan Qiu, Jiaming Yu, Yi Li, Fan Yang, Huiyuan Yu, Mengjuan Xue, Fan Zhang, Xin Jiang, Xueying Ji, Zhijun Bao

**Affiliations:** a Department of Gerontology, Huadong Hospital Affiliated to Fudan University, Shanghai, China; b Shanghai Key Laboratory of Clinical Geriatric Medicine, Shanghai, China; c Research Center on Aging and Medicine, Fudan University, Shanghai, China; d Department of Medical Oncology, Fudan University Shanghai Cancer Center, Shanghai, China; e Department of Oncology, Shanghai Medical College, Fudan University, Shanghai, China

**Keywords:** Gut microbiota, skeletal muscle, bile acid, FXR, FGF15/19

## Abstract

**Background:** Recent evidence indicates that host-gut microbiota crosstalk has nonnegligible effects on host skeletal muscle, yet gut microbiota-regulating mechanisms remain obscure.

**Methods:** C57BL/6 mice were treated with a cocktail of antibiotics (Abx) to depress gut microbiota for 4 weeks. The profiles of gut microbiota and microbial bile acids were measured by 16S rRNA sequencing and ultra-performance liquid chromatography (UPLC), respectively. We performed qPCR, western blot and ELISA assays in different tissue samples to evaluate FXR-FGF15/19 signaling.

**Results:** Abx treatment induced skeletal muscle atrophy in mice. These effects were associated with microbial dysbiosis and aberrant bile acid (BA) metabolism in intestine. Ileal farnesoid X receptor (FXR)-fibroblast growth factor 15 (FGF15) signaling was inhibited in response to microbial BA disturbance. Mechanistically, circulating FGF15 was decreased, which downregulated skeletal muscle protein synthesis through the extracellular-signal-regulated protein kinase 1/2 (ERK1/2) signaling pathway. Treating Abx mice with FGF19 (human FGF15 ortholog) partly reversed skeletal muscle loss.

**Conclusions:** These findings indicate that the BA-FXR-FGF15/19 axis acts as a regulator of gut microbiota to mediate host skeletal muscle.

## Introduction

Nowadays, the crosstalk between host and gut microbiota has been well recognised. Gut microbiota contributes to host metabolic homeostasis *via* gut-liver axis or gut-brain axis, while dysbiosis of gut microbiota could lead to various diseases including obesity, type 2 diabetes, non-alcoholic fatty liver disease and even mental disorders [1–5]. Recently, the notion of gut-muscle axis has gained accumulating attention, although the effect of gut microbiota on host skeletal muscle seems to be controversial. Several studies have found that lean body mass of germ-free (GF) mice and antibiotic-treated mice are increasing [6,7]. Whereas other studies found that skeletal muscle mass and function in these mice were impaired, and transplanting the gut microbiota from wild-type mice into GF mice resulted in an increase in skeletal muscle mass [8–11].

As the largest organ in the body, skeletal muscle is essential for maintaining the quality of life. Loss of muscle mass and function, which is a common consequence of sarcopenia, cancer cachexia and mitochondrial myopathies, can increase the risk of adverse outcomes including falls, fractures, disability and mortality [12–15]. Considering we currently lack specific drugs to treat skeletal muscle atrophy, our commensal partner gut microbiota is emerging as a promising therapeutic candidate. However, how they communicate and even regulate host skeletal muscle awaits further clarification.

Accumulating evidence suggests that gut microbiota could produce numerous bacteria metabolites to active different receptors in the host cells to contributes to host homeostasis. Bile acids (BAs) are important microbiota-host co-metabolites. The primary BAs are produced in the liver from cholesterol, after which they are conjugated with either glycine or taurine and released into the duodenum after a meal [16,17]. Conjugated primary BAs are deconjugated by bacteria containing bile salt hydrolase (BSH) and further metabolised into secondary BAs by gut microbiota modification [18,19]. Beyond their role in dietary lipid absorption, BAs are signalling molecules that regulate host glucose and lipid metabolism *via* binding to cellular BAs receptors, such as the nuclear farnesoid X receptor (FXR) [20–22]. Upon activation of ileal FXR, fibroblast growth factor 19 (FGF19, also called FGF15 in rodents) is produced, released into enterohepatic circulation, where FGF19 binds to hepatic FGF receptor 4/β-klotho (FGFR4/KLB) complex to regulate BAs synthesis [23,24]. Interestingly, previous studies have demonstrated that BAs, BAs receptors and BAs/FXR-induced FGF19 are associated with skeletal muscle mass and function [25–29].

Taken together, it is intriguing whether gut microbiota-BAs-FXR-FGF15/19 signalling is the key molecular machinery underneath the gut-muscle axis. Hence, to address this question, (1) we treated mice with a cocktail of antibiotics to extremely deplete gut microbiota, and further perturbed gut microbial BAs metabolism to investigate BA signalling; (2) we used FXR-induced FGF19 to evaluate its role in muscle regulation and found that it partly reversed depleted gut microbiota-induced skeletal muscle atrophy. These findings would shed light on the significance of gut microbiota in regulating host skeletal muscle mass and function *via* the BAs-FXR-FGF15/19 signalling.

## Method

### Mice and treatment

Nine-week-old male C57BL/6 mice (*N* = 32) were purchased from Shanghai Model Organisms Centre and maintained in specific pathogen-free and barrier conditions in Shanghai Laboratory Animal Research Centre. After arrival, mice were accommodated for 1 week. Mice were fed *ad libitum* and housed at 23 ± 1 °C with a relative humidity of 55 ± 5% under 12 h/12 h light/darkness cycle. The diet (20% of kJ from protein, 64% of kJ from carbohydrate, 16% of kJ from fat; D10012G, Research Diets) was identical for all groups. All the experimental protocols were approved by the ethical committee of School of Life Science, Fudan University.

At the age of 10 weeks, mice (*N* = 14) were randomly divided into control group (Veh mice, *N* = 7) and antibiotic-treated group (Abx mice, *N* = 7). A cocktail of five antibiotics were used to deplete the full spectrum of gut bacteria for 4 weeks, which is a standardised method used in numerous studies [30,31]. Among the antibiotics, ampicillin and vancomycin were used to deplete Gram positive strains, neomycin and metronidazole were used to deplete Gram negative aerobic and facultative strains, amphotericin-B was used to suppress fungal growth. Ampicillin (1 g/l) was administered in drinking water. Vancomycin (5 mg/ml), neomycin (10 mg/ml), metronidazole (10 mg/ml) and amphotericin-B (0.1 mg/ml) were dissolved in sterile water and administered daily by gavage. Gavage volume was adjusted to 10 ml/kg body weight. All the antibiotics were purchased from Yeasen Biotechnology (ampicillin, 60203ES10; vancomycin, 60213ES60; neomycin, 60207ES25; metronidazole, 60223ES25; amphotericin-B, 60238ES01).

Another group of 10-week-old male mice (*N* = 18) were randomly divided into vehicle- and phosphate buffered saline (PBS)-treated group (VP mice, *N* = 6), antibiotic- and PBS-treated group (AP mice, *N* = 6) and antibiotic- and FGF19-treated group (AF mice, *N* = 6). The same antibiotic treatment was given to AP group and AF group. VP group and AP group were treated with PBS/0.1% BSA solution (vehicle) by subcutaneous injection daily for 4 weeks. Because recombinant mouse FGF15 is unstable [32], and that bioactive FGF15 is not commercially available, AF group was treated with recombinant human FGF19 (969-FG, R&D Systems) subcutaneously (0.1 mg/kg body weight) in a PBS/0.1% BSA solution as previously reported [27].

### Grip strength test

The grip strength was measured three times for each mouse using a rodent grip strength metre (YLS-13A, Jinan Yiyan Company). The maximum value was used to represent the grip strength of each mouse.

### Body composition

After 4 h fasting (from 08:00 AM to 12:00 AM), mice body composition (fat and lean mass) was analysed by animal body composition analyser (EchoMRI). All the mice were carefully dissected after euthanasia by 10% chloral hydrate. The quadriceps, gastrocnemius, tibialis anterior, extensor digitorum longus and soleus muscles were dissected with little force tension and weighed. Liver was isolated and washed with ice-cold PBS. Ileum and colon were isolated and residual mesenteric fat tissue was resected. After that, ileum and colon were flushed with ice-cold PBS to clear faeces in the intestine. Caecum with caecum content were isolated and weighed, and then washed with ice-cold PBS. All the tissue were snap frozen at liquid nitrogen and stored at −80 °C for further analysis or immersed in 4% paraformaldehyde fix solution (PFA) for 24 h at 4 °C and then embedded in paraffin. Blood samples were gained from the eyeballs of mice, collected into tubes containing heparin and centrifuged for 30 min. Supernatants (plasma) were collected, snap frozen in liquid nitrogen and stored at −80 °C for further analysis.

### Immunolabelling assay

Muscle samples were dissected, immersed in 4% PFA and embedded in paraffin. 5-μm-thick tissue sections were stained with hematoxylin and eosin (H&E) for general morphological observations and were immunolabeled with anti-laminin antibody to quantify the muscle fibre size according to standard protocol. Anti-fast myosin skeletal heavy chain antibody and anti-slow skeletal myosin heavy chain antibody were used to recognize myofiber types. The images were acquired using OLYMPUS BX53 system (OLYMPUS). More than 200 myofibers from each mouse were analysed for quantification with ImageJ (NIH). Primary and secondary antibody information are listed in Table S1.

### Faecal DNA quantification and 16S rRNA sequencing analysis

Fresh faecal samples were collected from each mouse at the same time point (8:00 AM) weekly and then frozen in −80 °C until further investigation. The faeces were weighed and redissolved in working buffer to extract bacterial genomic DNA using QIAamp Fast DNA Stool Mini Kit (QIAGEN) according to the manufacturer’s instructions. Total bacteria load was evaluated by quantitative PCR (qPCR) using universal bacterial 16S ribosomal RNA (16S rRNA) primers F340, ACTCCTACGGGAGGCAGCAGT and R514, ATTACCGCGGCTGCTGGC as previously reported [33–35].

Faecal DNA of each mouse were subjected to PCR to amplify v3-v4 hypervariable region of the bacteria 16S rRNA gene using primers 338 F, ACTCCTACGGGAGGCAGCAG and 806 R, GGACTACHVGGGTWTCTAAT. 16S rRNA sequence tags were generated using Illumina Miseq system (Illumina) and were clustered to operational taxonomic unit (OTU) with 97% sequence similarity cut-off using UPARSE v.7.0 platform and taxonomically classified using Ribosomal Database Project (RDP) Classifier v.2.2 against the Silva (SSU123) 16S rRNA database with confidence threshold of 70% [36,37]. Alpha diversity, beta diversity and different species screening were analysed based on OTU with QIIME v.1.9.1. Further metagenome functional prediction of gut microbiota was analysed with PICRUSt2 algorithm [38].

### Bile acids measurement

BAs in caecum content, ileum, liver and colon were extracted using methanol containing internal standards and measured according to previously reported methods [34]. BAs in different tissues and BA synthesis marker 7-hydroxy-4-cholesten-3-one (C4) in plasma were analysed using ultra-performance liquid chromatography (UPLC)-triple quadrupole time-off-light mass spectrometry (Waters Corp) [34,39]. All the BAs standards and 7-hydroxy-4-cholesten-3-one (C4) standard were purchased from Sigma-Aldrich.

### Quantitative RT-PCR

Total RNA was extracted using Trizol reagent (Invitrogen). 1000 ng RNA was reversed to cDNA templates using PrimeScript RT Master Mix (Takara). Real-time PCR amplification and assay were performed using TB Green Premix Ex Taq (Takara) on StepOnePlus Real-Time PCR System (Applied Biosystems). Target gene expression levels were normalised to housekeeping gene *Rplp0*. Gene specific primers are listed in Table S2.

### Western blot

Proteins in ileum, liver and skeletal muscle were extracted with RIPA buffer containing protease inhibitors and phosphatase inhibitor and quantified with Pierce BCA protein assay kit (Thermo). Proteins were subjected to SDS-PAGE, transferred to PVDF membranes, and then respectively incubated with primary antibodies overnight at 4 °C. The blotting bands were illuminated by SuperSignal West Dura Extended Duration Substrate (Thermo) and captured with ChemiDoc XRS + System (BIO-RAD). The signal intensities of each antibody were quantified by ImageJ. Primary and secondary antibody information are listed in Table S1.

### FGF15 and FGF19 measurement

Mouse plasma FGF15 was determined by Mouse FGF15 ELISA Kit (detection sensitivity: 2.813 pg/ml; detection range: 4.688–300 pg/ml; XY-FGF15-Mu, Shanghai Xinyu Biotechnology). FGF19 was determined in mouse plasma by Human FGF19 ELISA Kit (detection sensitivity: 4.688 pg/ml; detection range: 7.813–500 pg/ml; XY-FGF19-Hu, Shanghai Xinyu Biotechnology) to verify that FGF19 could be enhanced by FGF19 injection. Experiment procedures were performed according to the manufacturer’s protocols.

### *In vivo* protein synthesis measurement

*In vivo* protein synthesis was measured by surface sensing of translation (SUnSET) method as previously reported [40–42]. After anaesthetizing, mice were intraperitoneal injected with0.040 μmol/g puromycin (Meilunbio) dissolved in 100 μl of PBS for 30 min. Then muscles were dissected for western blot analysis. Anti-puromycin antibody (MABE343, Millipore) was used to detect puromycin incorporation.

### Statistical analysis

Two-tailed Student’s *t*-test was used to evaluate differences between two groups, and the Mann–Whitney *U*-test was used for analysing data that were not normally distributed such as BAs measurement and 16S microbiome data. Multiple groups were analysed by one-way ANOVA followed by Fisher’s least significant difference (LSD) post hoc test. Values are expressed as mean ± SEM. *p* < .05 was considered to represent statistical significance.

## Results

**Figure 1.**
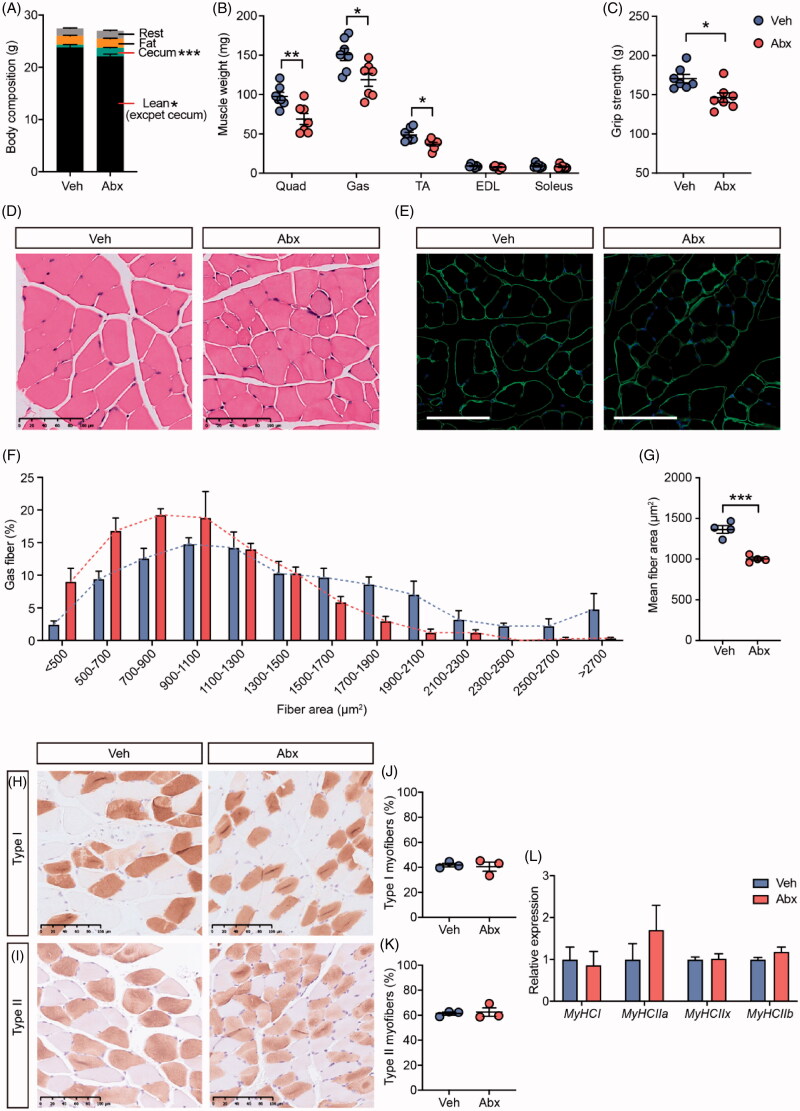
Effects of gut microbiota depletion on skeletal muscle phenotype. (A) Body composition of vehicle (Veh) mice and antibiotic-treated (Abx) mice. *N* = 7 per group. (B) Hind limb muscle weight of quadriceps (Quad), gastrocnemius (Gas), tibialis anterior (TA), extensor digitorum longus (EDL) and soleus muscles. *N* = 7 per group. (C) Grip strength. *N* = 7 per group. (D) H&E staining of gastrocnemius cross-sections. Scale bar = 100 µm. (E) Representative images of laminin-stained gastrocnemius muscle. Scale bar = 100 µm. (F) Cell size profiling of gastrocnemius fibre cross-sectional area (CSA). *N* = 4 per group. (G) Mean fibre area of (F). (H,I) Representative histology staining against slow skeletal myosin heavy chain (Type I) and fast myosin skeletal heavy chain (Type II) of soleus muscle. Scale bar = 100 µm. (J,K) Distribution of Type I and Type II myofibers in soleus muscle. *N* = 3 per group. (L) Expression of myosin heavy chain genes in soleus muscle measured by qRT-PCR. *N* = 7 per group. Data are presented as mean ± SEM. *p < .05, **p < .01, ***p < .001, Veh vs Abx. Student’s *t*-test.

To investigate whether gut microbiota could influence host skeletal muscle, we treated 10-week-old male mice with a cocktail of antibiotics for 4 weeks. Antibiotic treatment didn’t cause differences in body weight and food intake between antibiotic-treated mice (Abx mice) and vehicle-treated mice (Veh mice) (Figure S1A,B). We measured body composition of two groups of mice. Neither fat mass nor lean mass differed significantly between Veh mice and Abx mice (Figure S1C). However, caecum of Abx mice was significantly enlarged (Figure S1D and Figure 1(A)) as previously reported [30,31], so that the lean mass except caecum was decreased in the Abx group (Figure 1(A)). As the largest component of lean mass, skeletal muscle mass was reduced shown by hind limb muscles such as quadriceps, gastrocnemius and tibialis anterior (TA) muscles in Abx mice (Figure 1(B)). The grip strength of Abx mice was significantly weaker than that of Veh mice (Figure 1(C)). Thus, we further determined muscle fibre phenotype including myofiber size and myofiber type. On the one hand, H&E staining and immunofluorescence staining of gastrocnemius muscle sections detected that Abx mice showed a smaller myofiber size than Veh mice (Figure 1(D,E)). Quantitative morphometric analysis further revealed an increase in smaller myofibers as well as a decrease in larger myofibers with a consequently smaller mean fibre area in Abx mice compared with Veh mice (Figure 1(F,G)). On the other hand, immunohistochemistry for the slow myosin heavy chain (type I) and the fast myosin heavy chain (type II) showed there was no significant difference in the percentage of myofiber type (Figure 1(H–K)). Likewise, relative mRNA expression levels of myosin heavy chain genes were comparable in Veh mice and Abx mice (Figure 1(L)). Taken together, these results indicated that using a cocktail of antibiotics to delete gut microbiota in mice might lead to skeletal muscle atrophy in terms of fibre size but not fibre type.

**Figure 2.**
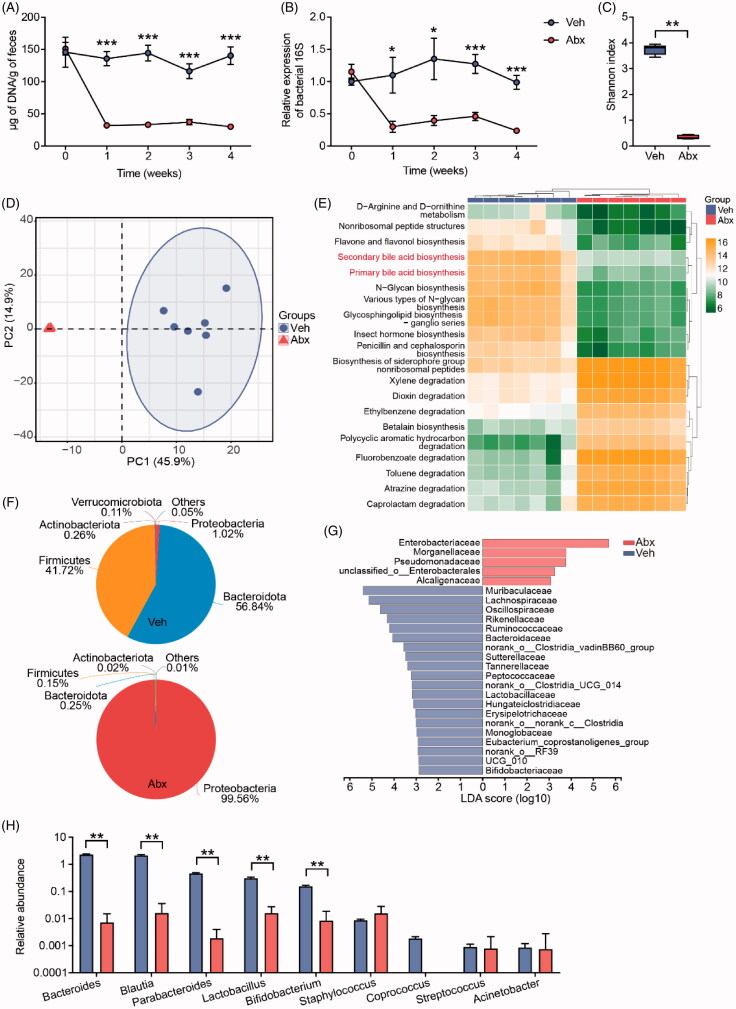
Antibiotic treatment decreased bacteria abundance and changed microbiota composition related to bile acid metabolism. (A) DNA content extracted from faeces of Veh and Abx mice. (B) Change of bacterial load in faeces from baseline, evaluated by qPCR using universal bacterial 16S rRNA primers. (C) Faecal bacterial alpha diversity using Shannon index. (D) Principal component analysis (PCA) plot based on OTU composition. Each dot represented an individual mouse. (E) Functional prediction of gut microbiota on microbial metabolism pathway using PICRUSt2 tool. (F) Gut microbiota composition of Veh and Abx mice at phylum level. (G) Gut microbiota composition at family level by Linear discriminant analysis effect size (LEfSe) analysis. (H) Top of BSHs containing bacteria stains at genus level. *N* = 7 per group. For (A, B and H), data are presented as mean ± SEM. For (C), the box plots indicate the median, 25th to 75th percentiles (boxes), and minimum to maximum values (whiskers). *p < .05, **p < .01, ***p < .001, Veh vs Abx. Student’s *t*-test or Mann–Whitney *U*-test.

We weekly collected fresh faeces pellets from mice and isolated faecal DNA. DNA contents in Abx mice faeces were significantly reduced compared with those in Veh mice faeces (Figure 2(A)). To exclude host DNA and fungal DNA, we assessed total bacterial load in the faeces of Veh mice and Abx mice by qPCR amplifying the bacterial 16S rRNA gene. The bacterial load was extremely depressed after antibiotic treatment (Figure 2(B)). We utilised sequencing of 16S rRNA from faecal DNA samples to investigate the global shift of gut microbiota in mice. Overall microbiota alpha- and beta-diversity of faecal DNA samples were compared at the OTU level. Antibiotic treatment strongly decreased microbial community diversity as measured by Shannon index (Figure 2(C)). Principal component analysis (PCA) showed that Abx mice clearly differed from Veh mice with a more homogeneous microbial population (Figure 2(D)). In addition to microbial profiling, we used PICRUSt [43], a computational tool to predict bacterial function, to assess the potential bacteria-related metabolism change. There were marked changes in microbial metabolism functions after antibiotic treatment (Figure 2(E)), including primary bile acid (BA) biosynthesis and secondary BA biosynthesis, which were both downregulated in Abx mice.

As a well-recognised microbiota-host co-metabolite, conjugated primary BAs are catalysed by bile salt hydrolase (BSH), an enzyme produced by bacteria, into unconjugated BAs, and further metabolised into secondary BAs by gut microbiota. We then focussed on whether the relative abundances of bacteria that can metabolise BA were changed after antibiotic treatment. At the phylum level, relative abundances of Bacteroidota, Firmicutes and Actinobacteriota, known phyla that harbour bacteria producing BSH [18,19], were dramatically decreased in the microbiome of Abx mice (Figure 2(F)). At the family level, the dominant taxa enriched in gut microbiota of Veh mice and Abx mice were significantly different (Figure 2(G)). Strains that have been reported to possess high BSH activity were the dominant families in Veh mice, such as Lachnospiraceae, Ruminococcaceae, Lactobacillaceae and Bifidobacteriaceae [19], while Enterobacteriaceae, Morganellaceae, Pseudomonadaceae, Enterobacterales and Alcaligenaceae were the dominant families in Abx mice. By comparing our data with other study’s database which reveals bacteria strains containing BSH at the genus level [19], we found that most of these bacteria were significantly decreased in Abx mice, such as *Lactobacillus* and *Bifidobacterium*, two famous probiotics that possess high BSH activity (Figure 2(H)) [44–46]. Taken together, antibiotic treatment strikingly deleted and disturbed gut microbiota in Abx mice, especially depressed bacteria exhibiting high BSH activity, so we inferred that intestinal BA metabolism was disturbed in Abx mice.

**Figure 3.**
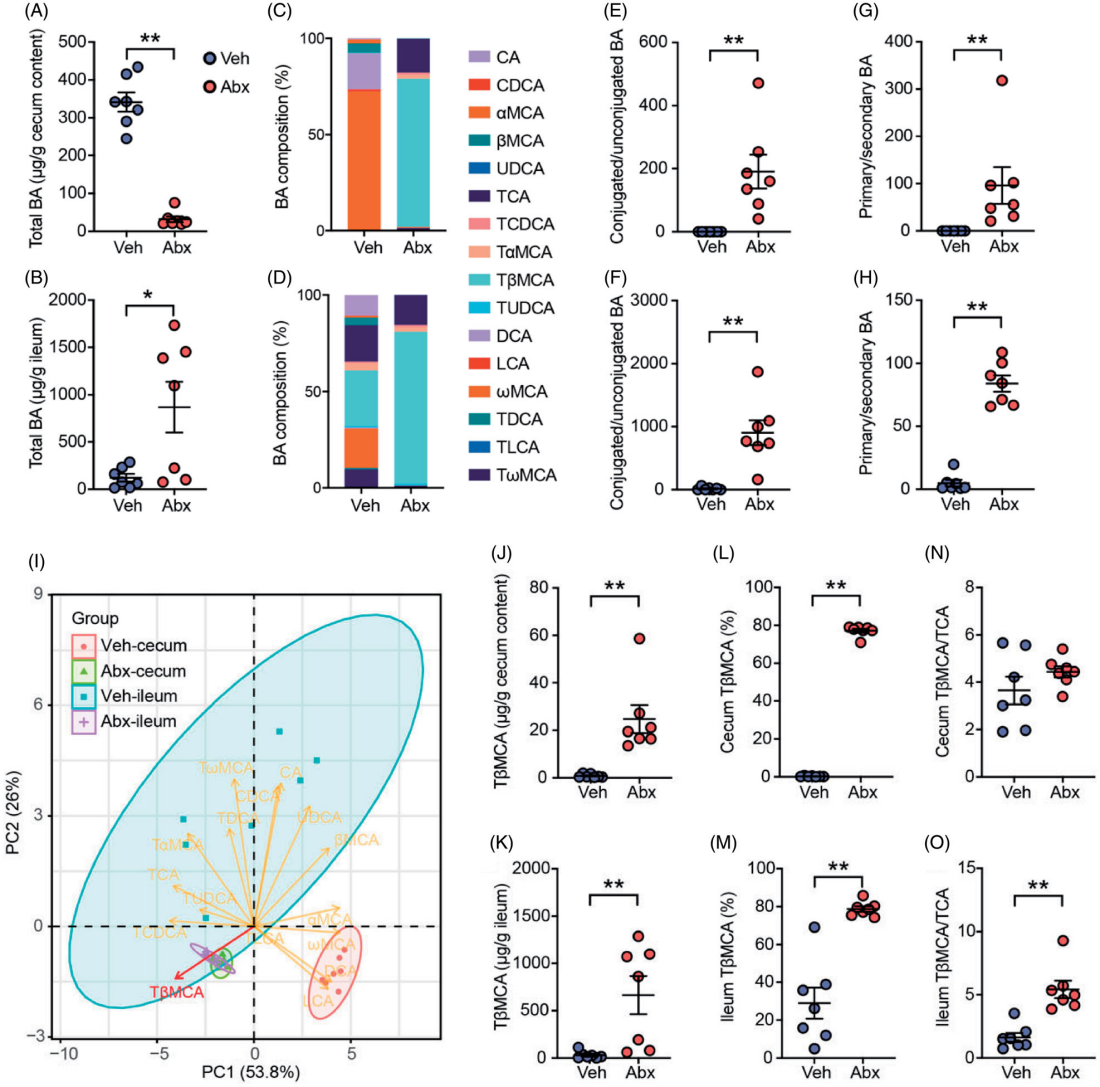
Depletion of gut microbiota inhibited microbial bile acid metabolism and induced TβMCA accumulation. Total BA content in caecum content (A) and ileum (B) of Veh and Abx mice. Stacked bar plot of BA composition in caecum content (C) and ileum (D). The ratio of conjugated/unconjugated BA in caecum content (E) and ileum (F). The ratio of primary/secondary BA in caecum content (G) and ileum (H). (I) PCA plot of BA in caecum content and ileum. Each dot represented an individual BA sample. Concentration of TβMCA in caecum content (J) and ileum (K). Percentage of TβMCA in caecum content (L) and ileum (M). The ratio of TβMCA/TCA in caecum content (N) and ileum (O). *N* = 7 per group. Data are presented as mean ± SEM. *p < .05, **p < .01, Veh vs Abx. Mann–Whitney *U*-test.

To further test whether the crosstalk between gut microbiota and host skeletal muscle depended on intestinal BA metabolism, we analysed BA profiles in caecum content and ileum tissue of two groups of mice. The BA pool size was significantly decreased in caecum but increased in ileum (Figure 3(A,B)), which suggested BA reabsorption was increased in Abx mice. The composition of BA profiles in caecum and ileum were obviously changed (Figure 3(C,D)). The ratios of conjugated/unconjugated BA and primary/secondary BA were significantly elevated in the caecum and ileum of Abx mice, which was a result of lacking high BSH activity bacteria (Figure 3(E–H)). PCA demonstrated a clear separation of caecum BA profile and ileum BA profile in Veh mice, whereas those of Abx mice were closer to each other (Figure 3(I)). This evidence suggested that deleting gut microbiota can strikingly change BA profiles, and this effect overrode tissue difference. Among all the BA species, tauro-β-muricholic acid (TβMCA) was the biggest contributor to this distinction. Different from other BA species, TβMCA is a well-recognised antagonist of BA receptor farnesoid X receptor (FXR) [47]. We found that both the concentration and proportion of TβMCA were significantly elevated in caecum and ileum of Abx mice (Figure 3(J–M)). In addition, the ratio of TβMCA/TCA, an indicator of FXR inactivation [34], was also increased after antibiotic treatment in ileum, although it was not significant different in caecum (Figure 3(N,O)). Thus, deleting gut microbiota was responsible for the alterations in microbial BA metabolism, particularly in accumulating the levels of FXR antagonist TβMCA.

**Figure 4.**
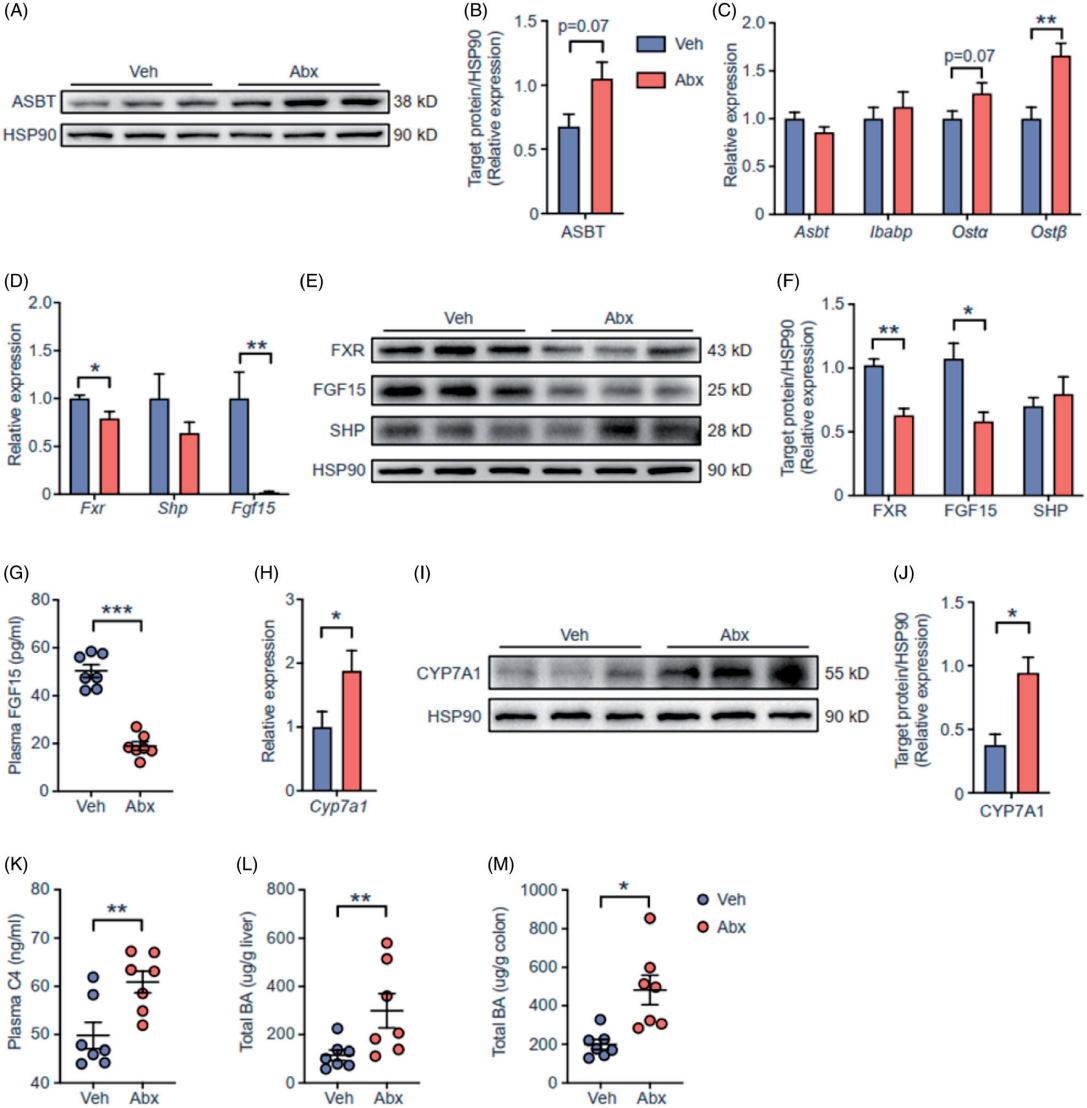
Depletion of gut microbiota inhibited FXR-FGF15 signalling in ileum. (A) The expression of ASBT in ileum were detected by western blot. HSP90 served as a loading control. (B) The statistical analyses result of the western blot of (A). (C) The relative mRNA levels of BA transporter genes in ileum from Veh and Abx mice. (D) The relative mRNA levels of *Fxr* and *Fxr* targeting genes in ileum. (E) The expression of FXR, FGF15 and SHP in ileum were detected by western blot. (F) The statistical analyses result of the western blot of (E). (G) Plasma FGF15 levels. (H) The relative mRNA levels of BA synthesis enzyme genes *Cyp7a1* in liver. (I) The expression of CYP7A1 in liver were detected by western blot. (J) The statistical analyses result of the western blot of (I). (K) Plasma 7-hydroxy-4-cholesten-3-one (C4) levels. (L,M) Total BA content in liver (L) and colon (M) of Veh and Abx mice. For (C,D, G,H and K–M), *N* = 7 per group. For (A,B, E,F and I,J), *N* = 3 per group. Data are presented as mean ± SEM. *p < .05, **p < .01, ***p < .001, Veh vs Abx. Student’s *t*-test.

We further sought to analyse BA-related signalling pathways in two groups of mice. The protein level of BA reabsorption gene solute carrier family 10, member 2 (*Asbt*) was increased in ileum of Abx mice (Figure 4(A,B)). BA transportation genes solute carrier family 51, alpha subunit (*Ostα*) and solute carrier family 51, beta subunit (*Ostβ*) were also upregulated in ileum of Abx mice (Figure 4(C)). Taken together, these data suggested BA reabsorption from intestinal lumen and secretion to portal blood circulation were increased in Abx mice, which might explain the changes of total BA content in Abx mice caecum and ileum (Figure 3(A,B)). In line with TβMCA accumulation in Abx mice intestine, ileal *Fxr* was inhibited at both mRNA and protein levels (Figure 4(D–F)). Interestingly, ileal FXR-targeting gene *Fgf15* was also inhibited but small heterodimer partner (*Shp*) remained unaltered in Abx mice. FGF15 (the mouse FGF19 ortholog) is a BA-FXR-induced enterokine secreted from distal ileum. It governs BA homeostasis by inhibiting bile acid synthesis enzymes such as cholesterol-7a-hydroxylase (*Cyp7a1*) in liver [23]. Plasma FGF15 was significantly decreased in Abx mice (Figure 4(G)), which consequently led to increasing *Cyp7a1* expression in Abx mice liver (Figure 4(H–J)). Further, plasma levels of the BA synthesis marker 7-hydroxy-4-cholesten-3-one (C4) were increased in Abx mice (Figure 4(K)), indicating BA synthesis was increased in Abx mice. The content of BA is not only increased in ileum (Figure 3(B)), but also increased in liver and colon of Abx mice (Figure 4(L,M)), and the gallbladder of Abx mice was enlarged (Figure S2A), indicating total BA pool size was increased after antibiotic treatment. *Cyp7a1* is also regulated by hepatic FXR-SHP signalling, but hepatic FXR signalling was comparable in Veh and Abx mice (Figure S2B-C). Taken together, deleting gut microbiota by antibiotic treatment altered microbial BA metabolism to remodel ileal FXR-FGF15 signalling.

**Figure 5.**
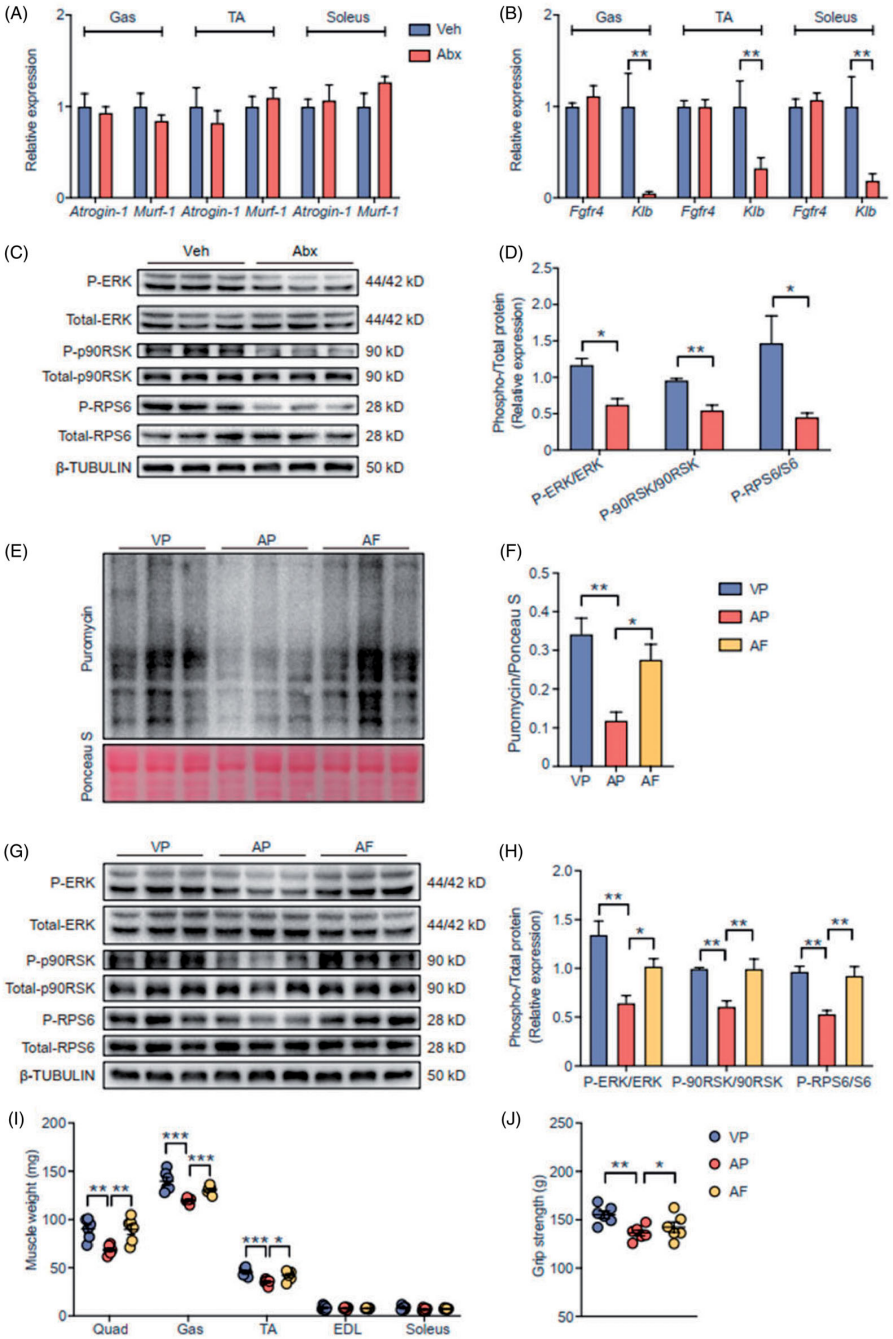
Microbiota-induced FGF15/19 regulated muscle protein synthesis *via* ERK signalling. (A) The relative mRNA levels of *Atrogin-1* and *Murf-1* in Hind limb muscles of Veh mice and Abx mice. (B) The relative mRNA levels of FGF15/19 receptor genes in Hind limb muscles. (C) The expression of phosphorylated proteins and their total proteins related to ERK signalling in gastrocnemius muscle. β-TUBULIN served as a loading control. (D) The statistical analyses result of the intensity of phosphorylated proteins relative to corresponding total proteins of (C). (E) Puromycin levels were detected by western blot to represent protein synthesis rate in gastrocnemius muscle of VP mice, AP mice and AF mice. Ponceaus S staining served as a loading control. (F) The statistical analyses result of the intensity of puromycin. Puromycin was normalised to Ponceaus S staining. (G) The expression of phosphorylated proteins and their total proteins in gastrocnemius muscle. (H) The statistical analyses result of the intensity of phosphorylated proteins relative to corresponding total proteins of (G). Hind limb muscle weight (I) and grip strength (J) of three groups mice. For (A,B), *N* = 7 per group. For (C–H), *N* = 3 per group. For (I,J), *N* = 6 per group. Data are presented as mean ± SEM. *p < .05, **p < .01, ***p < .001. Student’s *t*-test or one-way ANOVA.

Skeletal muscle mass depends on the balance between muscle protein synthesis (MPS) and muscle protein breakdown (MPB). We firstly found that muscle atrophy F-box (*Atrogin-1*) and muscle ring finger-1 (*Murf-1*), two well-known MPB-related genes expressed at comparable levels in muscles of Veh mice and Abx mice (Figure 5(A)). We then wondered whether MPS was changed in skeletal muscle of Abx mice, and whether the change of MPS was related to decreased circulating FGF15, because previous study has reported that FGF15/19 plays a novel role in skeletal muscle mass enlargement [27]. We assessed the mRNA levels of FGF15/19 receptor complex FGFR4/KLB in skeletal muscle. In accord with plasma FGF15 levels (Figure 4(E)), the mRNA level of *Klb* was significantly decreased in muscles of Abx mice, while that of *Fgfr4* was not significantly different between the two groups (Figure 5(B)). Extracellular signal-regulated protein kinase (ERK) signalling pathway, which is activated by FGF15/19 expression to catalyse protein synthesis to control muscle cell growth [32,48], was inhibited in skeletal muscle of Abx mice (Figure 5(C,D)). The phosphorylation of ERK1/2 with its downstream targets p90 ribosomal S6 kinase (p90RSK) and ribosomal protein S6 (RPS6) were decreased in skeletal muscle of Abx mice [49]. In addition to ERK signalling pathway, insulin signalling pathway also plays an essential role in MPS [50–52]. However, we found that the phosphorylation of insulin receptor β (INSR) and its targets protein kinase b (AKT), mechanistic target of rapamycin kinase (mTOR) and p70 ribosomal S6 kinase (p70S6K) were comparable in Veh and Abx mice (Figure S3A-B). Thus, we assumed that gut microbiota might mediate host MPS *via* FGF15/19-ERK signalling in an insulin independent way.

To further verify whether skeletal muscle atrophy in Abx mice was caused by decreased FGF15, another subset of mice were given the same antibiotic treatment with recombinant FGF19 injected subcutaneously for 4 weeks. The concentration of plasma FGF19 was undetectable in both vehicle- and PBS-treated mice (VP mice) and antibiotic- and PBS-treated mice (AP mice), but increased in antibiotic- and FGF19-treated mice (AF) mice (Figure S4). We used surface sensing of translation (SUnSET) method *in vivo* to estimate MPS rate in the three groups of mice [40–42]. Puromycin acts as a protein synthesis inhibitor that blocks protein translation through premature chain termination in the ribosome, so it becomes a biomarker to quantify MPS rate. Thirty minutes after puromycin injection, we detected puromycin in gastrocnemius muscle by western blotting using anti-puromycin antibody. Compared with VP mice, MPS rate, which was shown by puromycin-labelled peptides, was obviously decreased in AP mice, while MPS rate was significantly reversed in AF mice (Figure 5(E,F)). We also noted the phosphorylation of ERK1/2, p90RSK and RPS6 were reversed in AF mice compared with AP mice (Figure 5(G,H)). In line with these results, quadriceps, gastrocnemius and TA muscle mass and grip strength were increased in AF mice compared with AP mice (Figure 5(I,J)), but body weight was comparable among three groups (Figure S5). Collectively, we suggest that depleting gut microbiota disturbed gut microbiota-BA-FXR-FGF15/19-ERK signalling axis, which in turn decreased MPS, thereby inducing host skeletal muscle atrophy.

## Discussion

**Figure 6.**
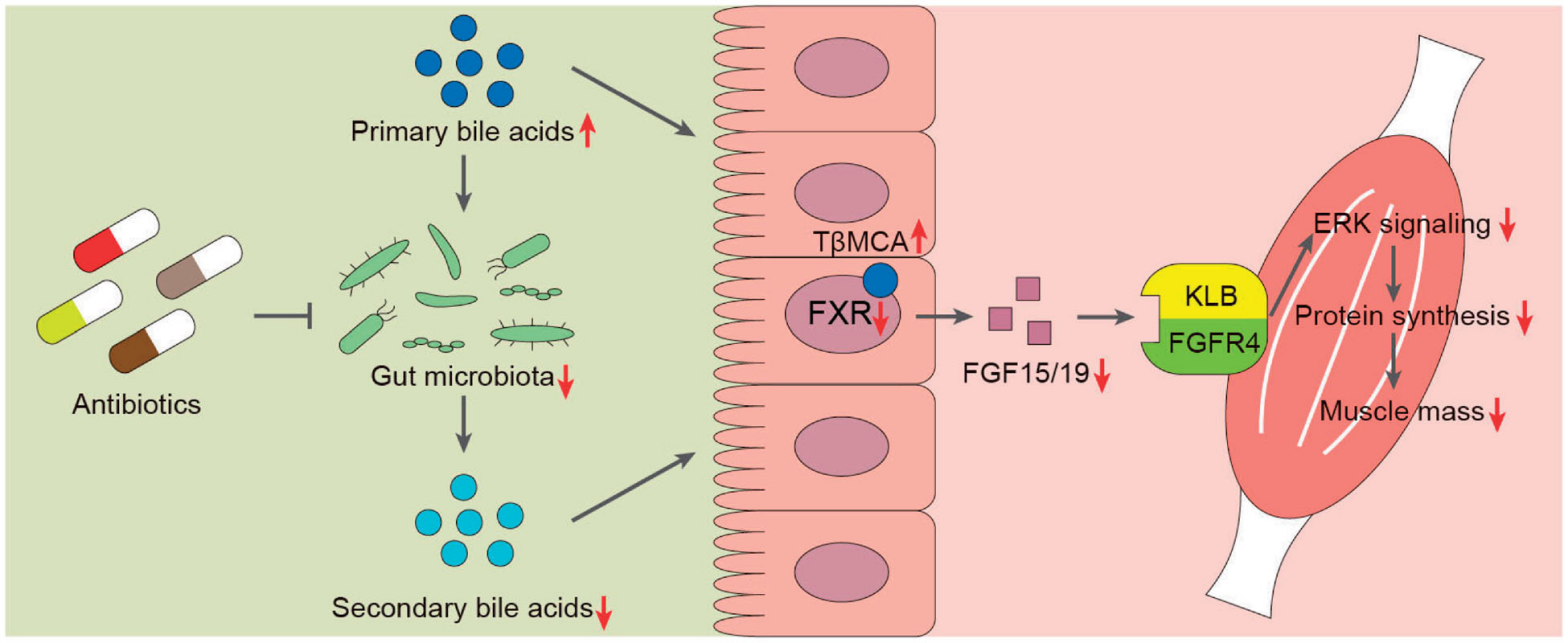
A proposed molecular mechanism of crosstalk between gut microbiota and host skeletal muscle *via* FXR-FGF15/19 signalling in mice.

How gut microbiota influences skeletal muscle mass remains elusive. In this study, we demonstrated that deleting gut microbiota by antibiotics can induce skeletal muscle atrophy in mice, resulting in altered body composition, reduced muscle mass and smaller myofibers size. As a result of microbial dysbiosis, which is characterised by lacking bacteria possessing BSH, the microbial metabolism of BA was strikingly obstructed so that conjugated primary BAs including TβMCA were increased. Ileal FXR-FGF15 signalling was subsequently inhibited because of FXR antagonist TβMCA accumulation. Finally, ERK signalling cascades were downregulated so that protein synthesis was impaired in muscle because of decreased circulating FGF15. Further FGF19 treatment at least in part reversed antibiotic-induced muscle loss in mice. These results suggested that the crosstalk between gut microbiota and host skeletal muscle might partly depend on BA-FXR-FGF15/19 signalling (Figure 6).

Our study confirms the results of earlier studies showing that gut microbiota plays an important role in the regulation of host skeletal muscle. Backhed team reported for the first time that compared with conventionalised mice, whole body lean mass was increased and fat mass was decreased in GF mice [6]. Another study also found that mice treated with antibiotics in early-life have significantly increased lean mass [7]. Skeletal muscle is the largest component of lean mass, so it is easy to conclude that host skeletal muscle mass would increase when gut microbiota was extremely depressed. However, our and other’s studies together found that gut microbiota depletion either in antibiotic-treated mice or GF mice can induce skeletal muscle atrophy [8–11]. In this study, body compositions were comparable in Veh and Abx mice, but caecum mass was significantly increased in Abx mice (Figure 1(A)), which is a typical characteristic of the absence of gut microbiota in GF and antibiotic-treated mice [30,31,53,54]. Therefore, we inferred that antibiotic treatment could cause skeletal muscle atrophy without influencing whole-body lean mass because of caecum enlargement.

The normal microbiomes in mice and humans are dominated by Firmicutes and Bacteroidetes, which harbour bacteria expressing BSH, a necessary microbial hydrolase that catalyses BA deconjugation. After antibiotic treatment, the Abx mice microbiome had a compositional shift to Proteobacteria and lacked bacteria stains expressing BSH. Previous data have reported that the elderly with high frailty scores, which indicate weak muscle performance, showed a significant reduction in the number of *Lactobacilli* and the number of Enterobacteriaceae was significantly higher in samples from very frail volunteers [55]. Feeding *ob*/*ob* mice with prebiotic induced significant changes in the gut microbiota, with *Lactobacilli* and *Bifidobacteria* being increased, thereby improving metabolic disorders with a higher muscle mass [56]. Directly feeding *Lactobacillus* and *Bifidobacterium* to aged mice resulted in increased muscle mass and grip strength [57], and this study also found these probiotics can change bile acid biosynthesis in the intestine. Similarly, our results also revealed that muscle-related bacteria that possess high BSH activity, especially *Bifidobacterium* and *Lactobacillus* [45], were decreased and Enterobacteriaceae became the dominant bacteria in Abx mice (Figure 2(G,H)). In sum, we suggested that the profiles of gut microbiota moved from a muscle-beneficial state towards a muscle-harmful state when mice were treated with antibiotics, which eventually induced skeletal muscle atrophy.

Recently, different studies found that the impacts of gut microbiota on host skeletal muscle were based on changing the expression of genes involved in the muscle peripheral circadian rhythm machinery [11], altering host glucose homeostasis regulation [9], and providing short-chain fatty acids (SCFAs) such as acetate and butyrate [8,10]. Lahiri team observed a trend of increased skeletal muscle mass in SCFAs-treated GF mice compared with untreated GF mice [8], another study also found that acetate treatment can increase endurance running time in antibiotic-treated mice [9]. These studies indicated that gut microbiota can use different microbial metabolites to regulate host skeletal muscle mass and function, which is related to multiple signalling pathways. Apart from their findings, our study firstly focussed on BA, another microbial metabolite, according to PICRUSt prediction and changes in microbial communities (Figure 2(E–H)). Antibiotic treatment successfully depressed bacteria to a very low level and thoroughly reshaped microbiota profiles so that microbial BA metabolism was destroyed. Because of lacking bacteria expressing BSH, conjugated primary BAs including TβMCA were increased but unconjugated BAs and secondary BAs were decreased in Abx mice. In line with the result of previous studies using GF mice or antibiotic-treated mice [47,58], we found that TβMCA was obviously increased in Abx mice intestine (Figure 3(J–M)). With the help of TβMCA, a well-known FXR antagonist, gut microbiota can regulate ileal FGF15 through an FXR-dependent pathway. In this study, the accumulation of ileal TβMCA (Figure 3(K,M)), inhibited ileal FXR-FGF15 signalling (Figure 4(D–F)) and decreased circulating FGF15 (Figure 4(G)) supported this finding.

Interestingly, previous studies found that FGF15/19 was an activator of protein synthesis in liver and skeletal muscle [27,32]. Treating mice with FGF19 can ameliorate muscle wasting in mice *via* ERK signalling, a pathway that regulates protein synthesis. We also revealed that muscle ERK signalling was inhibited by decreased FGF15 in Abx mice, independent of the insulin signalling pathway, which was also supported by a previous study [32]. In addition, SUnSET method confirmed that the MPS rate was decreased in antibiotic-treated mice. Treating Abx mice with FGF19 can reverse decreased MPS rate and increase muscle mass *via* FGF15/19-ERK pathway. Taken together, the impairment of protein synthesis in Abx mice muscles was associated with an inhibited FGF15/19 pathway.

Nevertheless, this study had several limitations of note. Firstly, GF mice were a better animal model to investigate gut microbiota-skeletal muscle axis. However, these mice not only have compensatory mechanisms to balance the absence of microbiota but also have a dramatically different bile acid profile [47]. Secondly, considering that FGF15/19 is a transversal metabolic coordinator at the crossroads of liver, skeletal muscle and intestine [59], we could not exclude the possibility that FGF15/19 exerted influence on the liver or intestine, which in turn regulated skeletal muscle. Therefore, skeletal-muscle-specific FGFR4/KLB knockout mice were better animal models to verify the effects of FGF15/19 on skeletal muscle in future studies. Thirdly, muricholic acids (MCAs), including TβMCA, are generally detected in rodents but not humans [47,60], therefore, whether the results can be generalised to human warrants further examination. Last but not least, several studies have reported that FGF15/19 contributed to hepatocellular carcinoma development [61,62], so the usage of FGF15/19 to ameliorate skeletal muscle atrophy needs more attention because of safety concerns. However, nontumorigenic FGF19 variants have been engineered and used to treat cholestatic liver disease and regulate glucose homeostasis [39,63,64]. Thus, it would be promising to investigate whether the FGF19 variant can treat skeletal muscle atrophy in future works.

In summary, we demonstrated for the first time that gut microbiota has a profound effect on mice skeletal muscle at least in part by BA-FXR-FGF15/19 signalling. On the one hand, antibiotics should be used with caution in clinical work, and whether antibiotic treatment induces skeletal muscle atrophy in human awaits further investigation. On the other hand, one inspiring discovery from our study is that bacteria-related FXR-FGF15/19 signalling could be potential targets for treating skeletal muscle atrophy.

## Supplementary Material

Supplemental Material

## Data Availability

The datasets are available from the corresponding author on reasonable request.
