# Supplementary material for: Depletion of gut microbiota induces skeletal muscle atrophy by FXR-FGF15/19 signalling
Source: Ann Med. 2021 Mar 30;53(1):508–22. doi: 10.1080/07853890.2021.1900593 (PMC8018554; doi:10.1080/07853890.2021.1900593)
Supplement: Supplemental Material [file IANN_A_1900593_SM5467.docx]

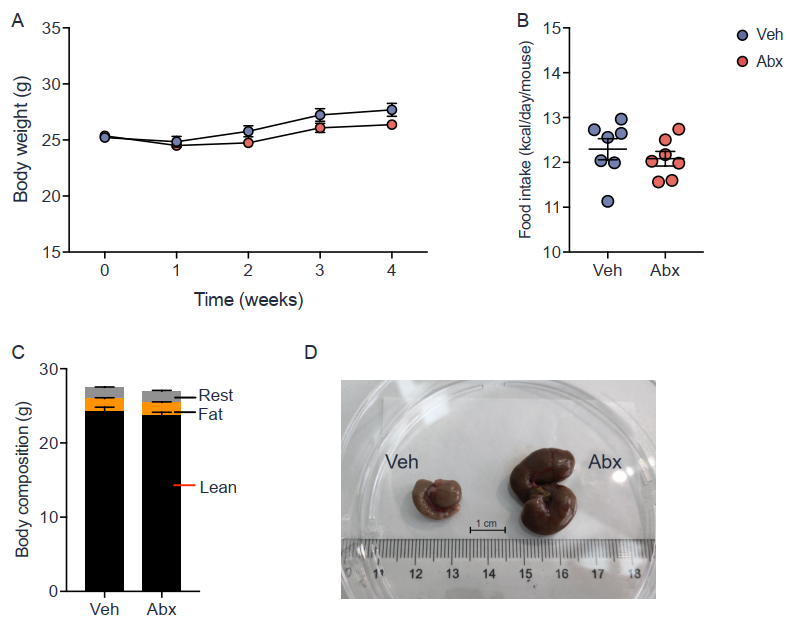


Figure S1. Other phenotypes in Veh mice and Abx mice. (A) The weekly body weight of Veh mice and Abx mice. N = 7 per group. (B) ﻿The daily food intake of two groups mice. N = 7 per group. (C) Body composition of two groups mice. N = 7 per group. (D) Representative images of cecum from Veh mice and Abx mice. ﻿Scale bar = 1 cm. Data are presented as mean ±SEM. Student’s t-test.


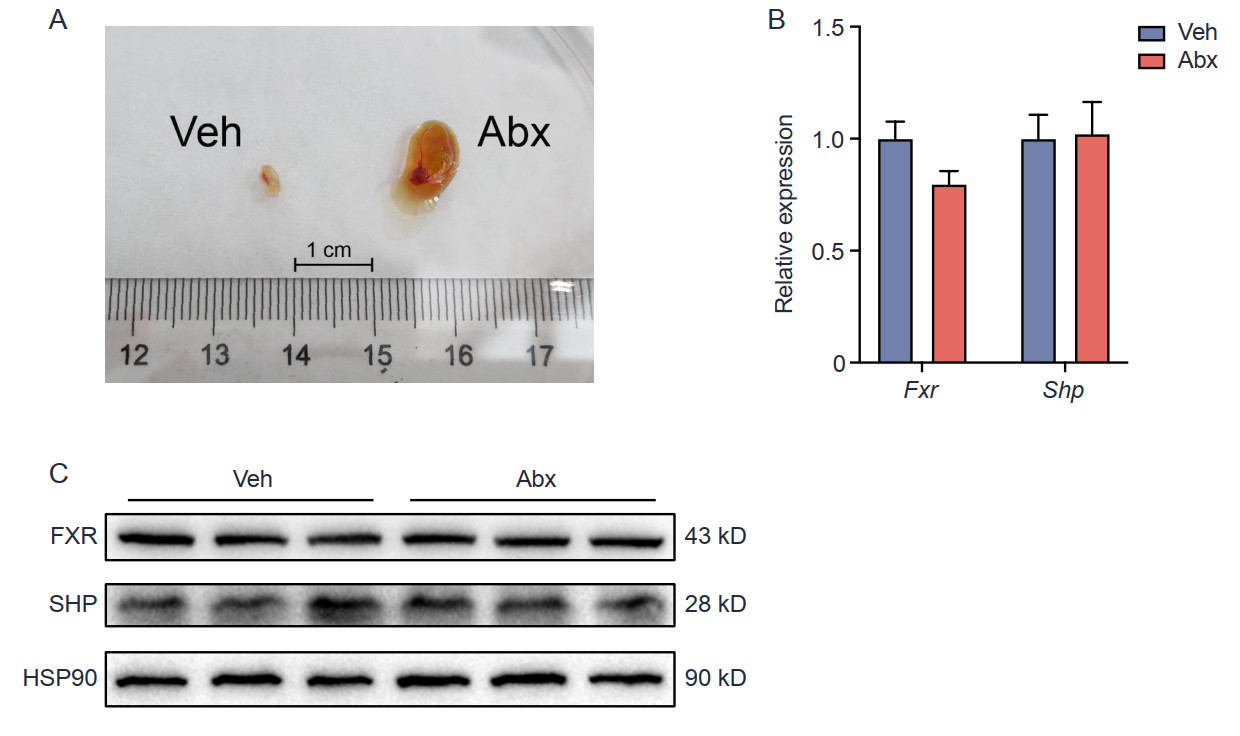


Figure S2. FXR signaling in liver of Veh mice and Abx mice. (A) Representative images of gallbladder from Veh mice and Abx mice. Scale bar = 1 cm. (B) The relative mRNA levels of *Fxr* and *Fxr* targeting gene *Shp* in liver. N = 7 per group. (C) The expression of FXR and SHP in liver were detected by western blot. N = 3 per group. Data are presented as mean ±SEM. Student’s t-test.


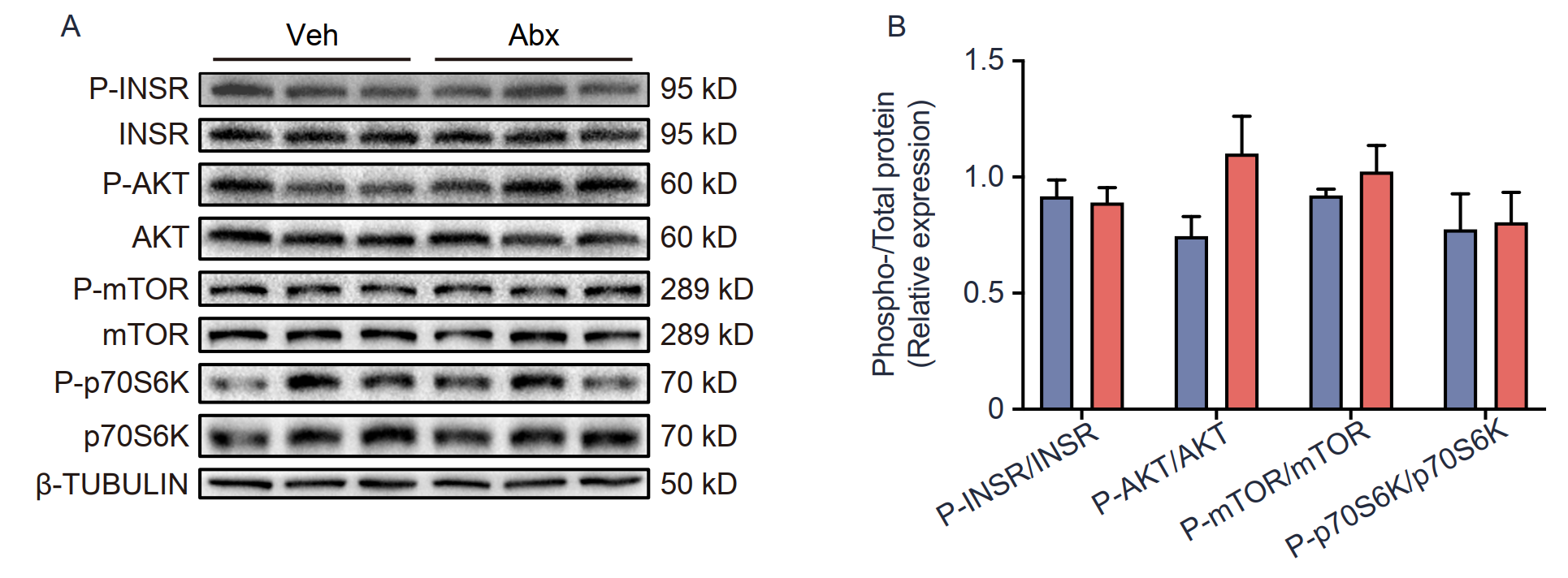


Figure S3. Insulin signaling in gastrocnemius muscle of Veh mice and Abx mice. (A) The expression of phosphorylated proteins and their total proteins related to insulin signaling pathway in gastrocnemius muscle. N = 3 per group. (B) The statistical analyses result of the intensity of phosphorylated proteins relative to corresponding total proteins of (A). Data are presented as mean ± SEM, one-way ANOVA.

Figure S4. Plasma FGF19 levels of three groups of mice. N.D., not detected. N = 6 per group. N.D., not detected. Data are presented as mean ± SEM.

Figure S5. The weekly body weight of Veh mice, Abx mice and FGF19 mice. N = 6 per group. Data are presented as mean ± SEM. one-way ANOVA.

Table S1. Antibody information.

| Antibody | Usage | Dilution | Source | Catalogue |
| --- | --- | --- | --- | --- |
| Rabbit anti-laminin | IF | 1:50 | Sigma-Aldrich | L9393 |
| Alexa Fluor 488 donkey anti-rabbit lgG (H+L) | IF | 1:400 | Life technologies | A21206 |
| Mouse anti-fast myosin skeletal heavy chain | IHC | 1:400 | Abcam | ab51263 |
| Rabbit anti-slow skeletal myosin heavy chain | IHC | 1:50 | Abcam | ab234431 |
| Goat Anti-Mouse IgG H&L (HRP) | IHC | 1:2000 | Abcam | ab205719 |
| Goat Anti-Rabbit IgG H&L (HRP) | IHC | 1:2000 | Abcam | ab205718 |
| Mouse anti-FXR | WB | 1:1000 | Santa Cruz | sc-25309 |
| Mouse anti-FGF15 | WB | 1:1000 | Santa Cruz | sc-398338 |
| Mouse anti-SHP | WB | 1:1000 | Santa Cruz | sc-271511 |
| Mouse anti-CYP7A1 | WB | 1:1000 | Santa Cruz | sc-518007 |
| Mouse anti-HSP90 | WB | 1:2000 | Santa Cruz | sc-13119 |
| Rabbit anti-RPS6 | WB | 1:1000 | Santa Cruz | sc-74459 |
| Rabbit anti-phospho-RPS6 | WB | 1:1000 | Santa Cruz | sc-293144 |
| Rabbit anti-ERK | WB | 1:2000 | Cell Signaling Technology | 4695 |
| Rabbit anti-phospho-ERK | WB | 1:2000 | Cell Signaling Technology | 4370 |
| Rabbit anti-mTOR | WB | 1:1000 | Cell Signaling Technology | 2983 |
| Rabbit anti-phospho-mTOR | WB | 1:1000 | Cell Signaling Technology | 5536 |
| Rabbit anti-p70S6K | WB | 1:1000 | Cell Signaling Technology | 2708 |
| Rabbit anti-phospho-p70S6K | WB | 1:1000 | Cell Signaling Technology | 9234 |
| Rabbit anti-p90RSK | WB | 1:1000 | Cell Signaling Technology | 9355 |
| Rabbit anti-phospho-p90RSK | WB | 1:1000 | Cell Signaling Technology | 9335 |
| Rabbit anti-INSR | WB | 1:1000 | Cell Signaling Technology | 3025 |
| Rabbit anti-phospho-INSR | WB | 1:1000 | Cell Signaling Technology | 3021 |
| Rabbit anti-AKT | WB | 1:1000 | Cell Signaling Technology | 4691 |
| Rabbit anti-phospho-AKT | WB | 1:1000 | Cell Signaling Technology | 4060 |
| Rabbit anti-ASBT | WB | 1:1000 | Proteintech | 25245-1-AP |
| Mouse anti-β-TUBULIN | WB | 1:1000 | ﻿Absin | ﻿abs830032 |
| Anti-rabbit IgG, HRP-linked Antibody | WB | 1:2000 | Cell Signaling Technology | 7074 |
| Anti-mouse IgG, HRP-linked Antibody | WB | 1:2000 | Cell Signaling Technology | 7076 |
|  |  |  |  |  |

Table S2. ﻿RT-PCR primers.

| Gene | Forward primer | Reverse primer |
| --- | --- | --- |
| *Asbt* | GTCTGTCCCCCAAATGCAACT | CACCCCATAGAAAACATCACCA |
| *Ibabp* | CTTCCAGGAGACGTGATTGAAA | CCTCCGAAGTCTGGTGATAGTTG |
| *Ostα* | TGTTCCAGGTGCTTGTCATCC | CCACTGTTAGCCAAGATGGAGAA |
| *Ostβ* | GTATTTTCGTGCAGAAGATGCG | TTTCTGTTTGCCAGGATGCTC |
| *Fxr* | TCCAGGGTTTCAGACACTGG | GCCGAACGAAGAAACATGG |
| *Shp* | TCTGCAGGTCGTCCGACTATTC | AGGCAGTGGCTGTGAGATGC |
| *Fgf15* | GCCATCAAGGACGTCAGCA | CTTCCTCCGAGTAGCGAATCAG |
| *Cyp7a1* | AGCAACTAAACAACCTGCCAGTACTA | GTCCGGATATTCAAGGATGCA |
| *Atrogin-1* | CAGCTTCGTGAGCGACCTC | GGCAGTCGAGAAGTCCAGTC |
| *Murf-1* | GTGTGAGGTGCCTACTTGCTC | GCTCAGTCTTCTGTCCTTGGA |
| *Fgfr4* | CAACTCCATCGGCCTTTCCT | CAGAACCAGTGAGCCTGATA |
| *Klb* | AGCCAATGGCATCGATGAC | ACACGCAGGACTTCTGTTCT |
| *Rplp0* | GAAACTGCTGCCTCACATCCG | GCTGGCACAGTGACCTCACACG |
